# Acute Sleep Deprivation Induces a Local Brain Transfer Information Increase in the Frontal Cortex in a Widespread Decrease Context

**DOI:** 10.3390/s16040540

**Published:** 2016-04-14

**Authors:** Joan F. Alonso, Sergio Romero, Miguel A. Mañanas, Marta Alcalá, Rosa M. Antonijoan, Sandra Giménez

**Affiliations:** 1 Biomedical Engineering Research Centre, Department of Automatic Control, Universitat Politècnica de Catalunya, Barcelona 08028, Spain; sergio.romero-lafuente@upc.edu (S.R.); miguel.angel.mananas@upc.edu (M.A.M.); mrtalcala@gmail.com (M.A.); 2 Barcelona College of Industrial Engineering, Universitat Politècnica de Catalunya, Barcelona 08037, Spain; 3 CIBER de Bioingeniería, Biomateriales y Nanomedicina (CIBER-BBN), Zaragoza 50018, Spain; 4 Drug Research Centre, Hospital de la Santa Creu i Sant Pau, Barcelona 08026, Spain; RAntonijoanA@santpau.cat (R.M.A.); SGimenez@santpau.cat (S.G.); 5 Department of Pharmacology and Therapeutics, Universitat Autònoma de Barcelona, Bellaterra 08193, Spain; 6 CIBER de Salud Mental (CIBERSAM), Madrid 28029, Spain; 7 Sleep Unit, Respiratory Department, Hospital de la Santa Creu i Sant Pau, Barcelona 08028, Spain

**Keywords:** prolonged wakefulness, sleep deprivation (SD), electroencephalography (EEG) transfer entropy (TE)

## Abstract

Sleep deprivation (SD) has adverse effects on mental and physical health, affecting the cognitive abilities and emotional states. Specifically, cognitive functions and alertness are known to decrease after SD. The aim of this work was to identify the directional information transfer after SD on scalp EEG signals using transfer entropy (TE). Using a robust methodology based on EEG recordings of 18 volunteers deprived from sleep for 36 h, TE and spectral analysis were performed to characterize EEG data acquired every 2 h. Correlation between connectivity measures and subjective somnolence was assessed. In general, TE showed medium- and long-range significant decreases originated at the occipital areas and directed towards different regions, which could be interpreted as the transfer of predictive information from parieto-occipital activity to the rest of the head. Simultaneously, short-range increases were obtained for the frontal areas, following a consistent and robust time course with significant maps after 20 h of sleep deprivation. Changes during sleep deprivation in brain network were measured effectively by TE, which showed increased local connectivity and diminished global integration. TE is an objective measure that could be used as a potential measure of sleep pressure and somnolence with the additional property of directed relationships.

## 1. Introduction

Sleep deprivation (SD) induces different negative alterations including behavioral, physical, metabolic, hormonal and mental changes among others [1,2,3], but the effects on cognitive activity are specially interesting. Specifically, acute total sleep deprivation reduces the function of several brain structures due to the decrease in the metabolic rate of the brain. Moreover, cognitive functioning and alertness are hypothesized to decrease after SD due to the reduced activity in the connections linking the cortex to the thalamus [4].

The most established theory to understand sleep regulation is the dual process model based on the coexistence of the homeostatic and the circadian processes [5]. While the circadian process is considered to act as a pacemaker for the control of sleep onset and offset, the homeostatic process is responsible for increasing the need for sleep as wakefulness continues. An objective monitoring of cerebral activity during SD can be easily obtained through the recording of electroencephalographic signals (EEG). Since the 80s, several studies have tried to model the circadian and homeostatic processes using EEG. For example, the slow-wave activity in non-rapid eye movement sleep (NREM), as measured by the power in the delta band, was assumed to reflect the homeostatic process during sleep [6]. A marker of sleep propensity in the waking EEG was proposed in the 90s after significant increases of EEG power in frequencies below 9 Hz, mainly in the theta band, were found during prolonged wakefulness [7,8,9]. Significant decreases of alpha power during resting state with eyes closed have also been reported after SD [10,11]. However, different works in the literature have pointed out that spectral power in the waking EEG can be influenced by the circadian process, suggesting that both wake duration and circadian modulation interact to modify the increase in theta power and, as a consequence, the theta power is not a monotonically increasing function [7,12,13]. In fact, both circadian and increasing homeostatic factors change simultaneously during prolonged wakefulness. EEG activity in the delta and theta bands is significantly affected by the combined effects of the circadian process and the increasing homeostatic pressure, as evidenced by forced desynchrony protocols [14]. Consequently, theta power better reflects sleep homeostasis during awake periods longer than 24 h, and therefore it cannot be considered an accurate measure of sleep pressure in shorter periods [13].

On the other hand, the relationship between the rise of theta activity during a 40 h prolonged wakefulness and the increase of slow-wave activity in the following sleep homeostasis during the first NREM episode was evidenced by a positive correlation between them [12]. Several studies in rodents have confirmed an increase of theta activity during sleep deprivation, showing changes in the spectral range probably as a function of the duration of the prolonged wakefulness, and a tendency for a similar positive correlation in humans, between the increase of theta power and subsequent slow-wave activity [13,15,16].

Previous studies have suggested alterations in brain connectivity after SD using EEG signals [17,18,19,20] and fMRI data [21,22,23]. Results have shown that prolonged wakefulness induces significant reductions of the efficient communication between brain regions, resulting in a loss of vigilance. Standard lineal methods such as cross-correlation and spectral coherence have revealed reduced EEG connectivity in theta, beta, and mainly in the alpha band after SD [24,25]. Likewise, techniques taking into account linear and nonlinear relationships, such as mutual information function and synchronization likelihood, have demonstrated that sustained wakefulness tends to attenuate EEG dependencies [19,26]. Recent studies applying graph theory to EEG data have shown that acute SD affects not only the local connectivity but also the functional brain network, bringing a more randomized network system as wakefulness continues [20,26]. On the other side, resting-state fMRI studies have shown focal reductions of functional connectivity of the default mode network (DMN), the brain network that is active at rest [22,27]. Finally, a recent fMRI study also has reported an unexpected increase of connectivity between the dorsal nexus and the prefrontal cortex after SD [21].

Transfer entropy (TE) was introduced by Schreiber [28] and Paluš [29] and constitutes a model-free measure of directed information that quantifies the reduction of uncertainty in inferring the future state of a process when the current and past states of another process are known. TE is strictly a measure of the predictive information transferred between processes, and in the neuroscience context may be interpreted as information transferred in service of a distributed computation [30]. TE has been successfully applied to quantify EEG directed connectivity for measuring unconsciousness in anesthesiology [31,32], for studying alterations of connectivity caused by different neurological disorders [33,34], or for assessing pharmacological effects on the brain [35,36]. TE has also been applied to several other fields ranging from ecology to economy, and provides a useful measure to understand complex systems that can be viewed as a network of interacting processes [30].

The aim of this work was to identify and evaluate both linear and nonlinear information transfer after SD. Additionally, the consistency of a connectivity-based measure was assessed as a possible marker of the sleep propensity. Using a robust methodology based on recordings of sleep-deprived volunteers, EEG acquisitions spaced 2 h over a total time of 36 h were performed and TE was applied to assess directional information exchange between cerebral regions during prolonged wakefulness.

## 2. Materials and Methods

### 2.1. Design of the Study

Eighteen healthy adult volunteers (mean age 28 years, ranging from 21 to 34) were included in the study. All participants underwent physical examination, ECG, and standard laboratory tests so that they were confirmed to be in good health. They were not allowed to take any medication during the study without supervision of the investigators and they kept regular sleep-wake habits the month before the study initiation and during the study. Individual sleep diaries were checked to confirm that sleep hygiene was correctly maintained.

**Figure 1:**
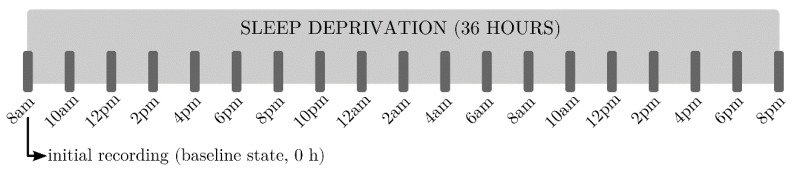
Schematic description of the experimental protocol for EEG recording.

The study was conducted in accordance with the Declaration of Helsinki and subsequent revisions concerning experimentation in humans. Volunteers gave written informed consent before the start of the study and were paid for their participation. The study was designed according to an intervention-controlled and crossover scheme. All volunteers completed the study, consisting in a sleep deprivation experimental session of 36 h after arrival at 7:30 am. An initial EEG recording was acquired at 8 am as baseline, and successive EEG registers were recorded every 2 h. Volunteers were continuously supervised to prevent them from snoozing. Figure 1 shows the schematic diagram of the experimental trial, in which subjects underwent EEG recordings and somnolence scale tests every 2 h until the end of the experiment.

The subjective measure of somnolence used was a visual analogue scale (VAS). A VAS is a 100 mm long line where subjects draw with a pen the point of the line that reflects better their current state between two extremes (sleepy and alert, in this study). VAS scores have proven to be very sensitive to partial and total sleep deprivation, and also to the time of the day [37,38,39].

### 2.2. EEG Acquisition and Preprocessing

Spontaneous EEG and EOG signals with eyes closed were acquired at a sample rate of 250 Hz during 3-min periods by a NeuroScan SynAmps amplifier (CompumedicsNeuroscan, Charlotte, NC, USA) using a band-pass filter with cutoff frequencies set to 0.1 and 45 Hz. The experimenter kept the volunteers alert by gentle acoustic stimulation as soon as drowsiness patterns appeared in the recorded signals. Two EOG (vertical and horizontal) and 19 scalp EEG signals were acquired by means of electrodes placed according to the international 10/20 system (Fp1/2, F3/4, Fz, F7/8, C3/4, Cz, T3/4, T5/6, P3/4, Pz, and O1/2). Signals were digitally resampled to 100 Hz and underwent artifact reduction and epoch rejection procedures.

The first consisted of a two-step artifact reduction process: a stage of ocular artifact reduction followed by automatic artifact rejection. The reduction of ocular contamination was based on blind source separation, a signal processing technique whose goal is to express a set of signals as a linear combination of statistically independent component signals. For this purpose, the SOBI algorithm [40] was used, which is based on eigenvalue decomposition of a linear combination of several time-delayed covariance matrices. Corrected EEG signals were reconstructed from the remaining components, by zeroing out the rows of the decomposition matrix corresponding to the ocular sources. The automatic identification of these ocular sources was based on spectral features and scalp topography as described in [41].

Secondly, the automatic artifact rejection procedure based on time- and frequency-domain approaches [42] was applied to each 5-s epoch available from the original 3-min recording. Any epoch breaking one of these rules was considered as artifacted:

1. Maximum amplitude lower than ±150 mV to avoid electrode-related artifacts or remaining ocular contamination.
2. Absolute power in the 35 to 45 Hz band lower than 25 mV^2^ in each EEG channel in order to reject muscular artifacts. Frontopolar, frontal and temporal derivations had a higher threshold set to 50 mV^2^.
3. Absolute power ratio of alpha (7.5 to 13 Hz) to delta (0.5 to 3.5 Hz) power higher than a variable threshold depending on the channel amplitude and global alpha activity present in the signal. This criterion was set to counterbalance a possible incomplete elimination of ocular artifacts, mainly indicated by an increase in delta activity.

After computing the two-step artifact processing procedure, an almost artifact-free 60-s segment (12 consecutive epochs) was cropped out and zero-phase filtered between 0.5 and 35 Hz using a type II Chebyshev filter of order 8.

### 2.3. Spectral Analysis

The literature reports several spectral EEG measures related to sleep propensity and homeostasis. Increased theta power, as well as reduced alpha power and coherence have been described [7,8,9,12]. Also, the ratio of alpha to delta and theta has been considered a possible indicator of the alertness of the subjects [43]. Hence, delta, theta and alpha powers were calculated, as well as the magnitude squared coherence (*MSC*) in the alpha band, deemed as an electrophysiological index of the arousal level in humans [24,25]. Spectral estimation was performed using the Welch periodogram with 5-s Hanning windows and 25% of overlapping. Powers were obtained by integrating the frequency ranges of interest (0.5 to 3.5 Hz for delta, 3.5 to 7.5 Hz for theta, and 7.5 to 13 Hz for alpha). Although there are some discrepancies in the literature about the frequency boundaries of the EEG bands, the proposed spectral ranges are similar and partially overlap with the bands used in prolonged wakefulness studies [10,11,12,44]. The ratio of alpha power to slow frequencies (delta and theta) was used as an objective measure of alertness [43,45]. *MSC* was obtained according to the following equation:

(1) $$MSC(f)=\frac{|S_{xy}(f){|}^{2}}{S_{xx}(f)S_{yy}(f)}$$

where *S_xx_* indicates the autospectral density of a signal *x*, and *S_xy_* the cross-spectral density of two signals *x* and *y*. The coherence in the alpha band <*MSC*_α_> was calculated for all 171 possible pairs of EEG channels by averaging the *MSC* function in the corresponding frequency range, and was obtained as an indicator of the arousal level.

### 2.4. Transfer Entropy Analysis

Transfer entropy (TE) measures the amount of predictive information between two signals and is defined as:

(2) $$\begin{array}{lll} {TE}_{y\to x} & = & \sum_{x_{n+1},x_{n},y_{n}}p\left(x_{n+1},x_{n},y_{n}\right)log\left(\frac{p\left(x_{n+1}|x_{n},y_{n}\right)}{p\left(x_{n+1}|x_{n}\right)}\right)\\ & = & \sum_{x_{n+1},x_{n},y_{n}}p\left(x_{n+1},x_{n},y_{n}\right)log\left(\frac{p\left(x_{n+1},x_{n},y_{n}\right)p\left(x_{n}\right)}{p\left(x_{n},y_{n}\right)p\left(x_{n+1},x_{n}\right)}\right) \end{array}$$

where *x* and *y* represent the signals under study, and *n* indicates the sample time. *TE* is an inherently non-symmetric measure (as opposed to *MSC*, for example) and thus 342 connections were introduced in the analysis. TE was calculated using a nonparametric methodology based on equiquantal binning, that is, estimating probability distributions using marginal equiquantization and histogram estimation. This procedure has proven to be effective for real data when sufficient data points (in practice, a few thousands) are available [46,47].

### 2.5. Statistical Analysis

All variables at each recording time were compared to the baseline using two-sided Wilcoxon signed-rank tests to evaluate the course of changes induced by sleep deprivation. Statistically significant changes indicated by individual tests were represented using schematic representations of the scalp: for spectral variables, topographic maps representing significant changes at each electrode location; for connectivity variables, maps featuring lines connecting pairs of electrodes whose connectivity changed significantly with respect to the baseline.

In each of these maps, and especially for connectivity maps, several statistical comparisons were performed. An omnibus test based on the binomial theorem was used as a multiple correction procedure to detect significant connectivity maps [48]. Omnibus tests have been widely used in several fields such as neurology and pharmacology [49,50,51,52] and allow the use of uncorrected probability values, but the multiple comparison issue can be solved by only indicating a significant map when the number of significant differences that it contains exceeds the threshold imposed by the omnibus test.

In each map warm colors indicated increases of the variable with respect to the baseline, whereas cold colors indicated decreases. Color intensity and line thickness were related to the associated probability value of significant differences as described in Table 1.

In addition to statistical representations, and given that TE is a directional measure, additional separate maps were used to represent sources and sinks of information transfer (green and gray shaded, respectively). In these maps, the darkest shade represented seven or more connections going in or out of the corresponding electrode, whereas the white color indicated no activity.

Finally, data were explored for correlations between TE alterations and changes in spectral variables (theta power and alpha coherence) and objective and subjective indexes of somnolence (ratio alpha/slow and VAS). Three brain regions of interest (anterior, posterior and global) were considered by averaging TE changes corresponding to all the possible pairs of sensors inside each region examined. Correlations were calculated using Pearson’s correlation coefficient. Only significant effects were considered (*p* < 0.05).

## 3. Results

**Figure 2:**
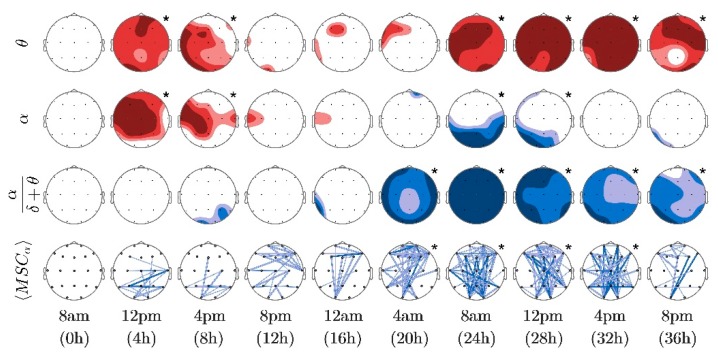
Time course of significant changes induced by prolonged wakefulness on spectral variables and magnitude squared coherence. From top to bottom, each row corresponds to θ power, α power, ratio α/(δ + θ) and α coherence, respectively. Although all EEG recordings performed every 2 h were analyzed, only time-points spaced 4 h are depicted. Increases and decreases of spectral variables with respect to baseline (0 h) are indicated with hot and cold colors as indicated in Section 2.5 and Table 1. Significant changes in coherence are indicated by connections between electrodes that follow the same color code. Significant maps, according to the binomial omnibus test, are indicated by an asterisk.

Volunteers showed significant theta power increases, as seen in Figure 2, especially after 24 h of sleep deprivation. Figure 2 also shows the time course of alpha power, related to a relaxed state while awake with eyes closed, which was considerably reduced at the occipital locations after 24 h. Specifically, the alpha decrease was significant at 24 h and 28 h, but not at 32 h and 36 h. In a similar manner, an increase in theta was observed at 4 h and 8 h, then disappeared temporarily and emerged again after 24 h of prolonged wakefulness. This response is consistent with previous findings, potentially reflecting the superposition of the homeostatic increase and the effect of the circadian regulation. Moreover, the ratio of alpha to slow frequencies, which is considered a good indicator of the state of alertness, was also significantly reduced after 20 h of prolonged wakefulness. The bottom row of Figure 2 depicts the changes obtained from alpha coherence. Significant maps were obtained after 20 h, indicating a state of reduced arousal.

**Figure 3:**
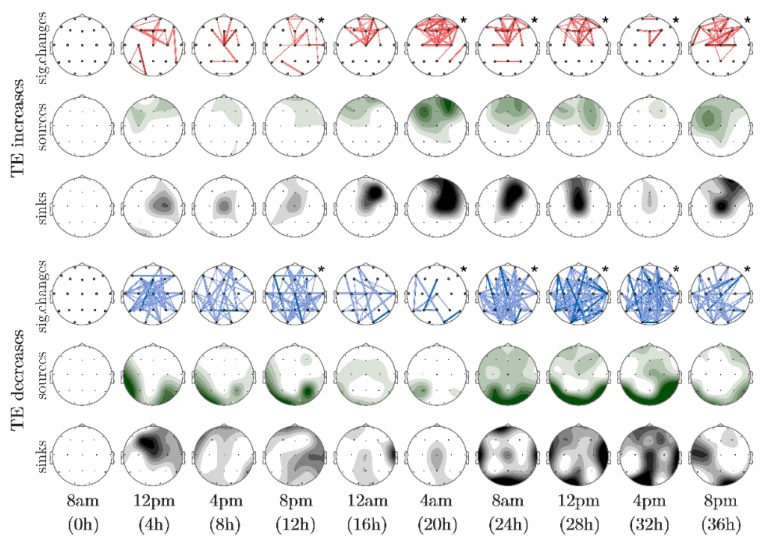
Time course of significant changes induced by prolonged wakefulness on transfer entropy. The top three rows correspond to TE increases and the bottom three to TE decreases, as indicated by hot and cold colors of the lines in the connectivity maps, respectively. In addition to increases and decreases with respect to baseline (0 h), topographical representations of sources and sinks of TE are shown, drawn in green and black shades respectively. The darkest shades indicate 7 or more significant changes of TE at the corresponding electrode. Although all EEG recordings performed every 2 h were analyzed, only time-points spaced 4 h are depicted. Significant maps, according to the binomial omnibus test, are indicated by an asterisk.

With respect to TE, an interesting course of the obtained changes was observed (Figure 3). The whole-brain averages in each time point show a reduction of information transfer, especially after 24 h of sleep deprivation.

Figure 3 depicts the origin of these TE reductions as parieto-occipital, affecting several frontal, parietal, temporal and occipital locations. Interestingly, TE also evidenced significant increases located in the anterior region, not evident in the average trend, peaking after 20 h of prolonged wakefulness and mainly with frontal origins and destinations.

**Figure 4:**
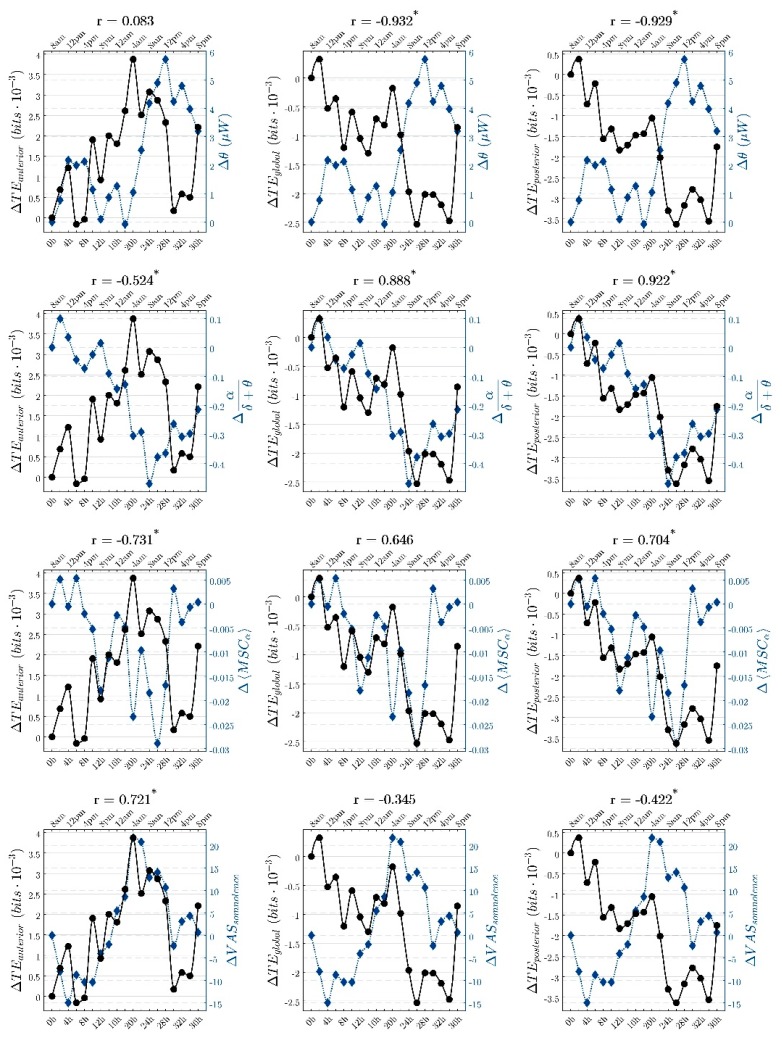
Time course and Pearson correlation coefficients between changes in TE (continuous black traces and left vertical axes in all graphs) and spectral variables, coherence and subjective somnolence (blue dotted traces and right blue axes in all graphs). Horizontal labels indicate the time of the day (**top**) and the time spent since the start of the experiment (**bottom**). The change in average TE was evaluated in three regions, shown from left to right column, respectively: anterior (all possible pairs of channels in the frontopolar and frontal locations), whole head (all possible pairs of channels), and posterior (all pairs in the occipital, parieto-occipital and temporal locations). From top to bottom, each row depicts the comparison of TE to θ power, ratio α/(δ + θ), α coherence, and subjective somnolence measured by visual analogue scales, as indicated by the blue labels to the right of each graph. Pearson correlation coefficients are indicated on top of each graph, and an asterisk indicates those coefficients that were significant.

Figure 4 presents the temporal course of average TE, normalized with respect to the baseline, for all subjects. Three different TE values were considered: average TE in the anterior region (*TE_anterior_*, corresponding to fronto-polar and frontal electrodes), average global TE (*TE_global_*), and average TE in the posterior region (*TE_posterior_*, corresponding to occipital, parieto-occipital and temporal electrodes). Indices related to sleep pressure during prolonged wakefulness, state of alertness and arousal, and a subjective variable of somnolence, which had been previously reported, are also presented.

As seen in the traces of TE_global_ and TE_posterior_, TE decreases exhibited the combined effect of both the circadian and the increasing homeostatic processes. Pearson correlation coefficients shown above each graph indicate that *TE_global_* and *TE_posterior_* were highly anticorrelated with theta power and highly correlated with the ratio of alpha to slow bands, showing that TE decreases were mainly associated with the circadian modulation at the beginning and later were able to follow the increased sleep pressure and the reduced alertness, especially after a prolonged period of sustained wakefulness (longer than 24 h). Lower coefficients were obtained between these two TE variables and the subjective somnolence.

On the other hand, TE increases located mainly in the frontal region did not seem to follow the circadian modulation because of the very low correlation with theta power. Interestingly, *TE_anterior_* exhibited the strongest (negative) correlation with the subjective somnolence, showing that TE increases could serve as an objective measure of the subjective drowsiness experienced by volunteers.

## 4. Discussion

Sleep deprivation induces known effects on the spectral content of eyes-closed spontaneous EEG signals such as a decrease of alpha power and coherence [10,11,25,26,53,54], but also an increase in theta power, which has been considered a useful measure of accumulated sleep pressure during long periods of sustained wakefulness [7,8,9,12,26]. Low frequency power changes are most apparent in frontal brain areas, indicating that the prefrontal cortex may be especially susceptible to the effects of prolonged wakefulness [55]. In this sense, the results obtained in this study using spectral variables and coherence are in agreement with all previously published works. Despite the slight differences in defining the frequency boundaries of the spectral bands with respect to the literature [9,10,11,12,44,56], known findings during sleep deprivation were reproduced: increases in theta activity and decreases in alpha power and coherence [7,8,9,12,24].

The effects of prolonged wakefulness on cognitive and behavioral performance have been extensively studied, showing different alterations depending on the brain area considered and the mental task performed. For example, the activity of the temporal lobe, associated with language processing, is mainly suppressed during verbal learning tasks [57]. Similarly, sleep deprived subjects solving mathematical problems do not show activity in the parietal lobe, which is the corresponding responsible brain area [58]. However, the prefrontal cortex exhibits more activation during sleep deprivation during a divided attention task combining arithmetic and verbal learning [58]. The prefrontal cortex plays an important role in executive functions, selective attention, and short-term memory maintenance [59].

Several fMRI studies have shown reductions of functional connectivity of the default mode network (DMN) nodes after prolonged wakefulness reflecting the increase of sleep propensity [22,27,60]. The DMN is an interconnected set of brain regions that includes cortical midline structures such as the medial frontal cortex, the precuneus and the inferior parietal lobule, and it is moderately activated during wakeful rest, attenuated during deep sleep, and shows reduced functional connectivity after prolonged wakefulness. A recent study confirmed the disturbed connectivity pattern of the DMN after SD, indeed revealing an increase of functional connectivity between the dorsal nexus and different areas of the dorsolateral prefrontal cortex [21]. An increased prefrontal sensitivity to cognitive requests has also been found after SD [58,61]. Different hypotheses can be used to interpret the SD-related increase of prefrontal activity: it might be a compensatory mechanism of cerebral activation that neutralizes deactivations in other regions to maintain conscious awareness [61,62], or a possible SD-related reactivation of top-down control on negative emotional processing [63,64].

Transfer entropy (TE), a directed functional connectivity measure, was applied in this study in order to identify information transfer during sleep deprivation, enabling the assessment of directed interactions. TE is a recent technique which has been successfully applied to assess brain connectivity in neurological disorders [34,65,66] and in neuropharmacology [32,36]. In general, during sleep deprivation TE showed medium- and long-range significant decreases originated at the occipital areas and directed towards different regions, which could be interpreted as the transfer of predictive information from parieto-occipital activity to the rest of the head. At the same time, short-range increases were obtained over the frontal areas, following a consistent and robust time course with significant maps after 20 h of prolonged wakefulness. These electrophysiological findings are in agreement with the changes found in the topology of brain networks after sleep deprivation, hypothesized due to a loss of optimal configuration for information processing, that is, increased local connectivity and diminished global integration, which are restored during sleep [26]. Moreover, differences in TE could be related with previous data showing that sleep deprivation might elicit an imbalance of midline posterior and anterior regions of the DMN in the following wake [60,67].

It is known that sleep pressure increases progressively during waking and declines during sleep, modeled by the exponential decay of slow-wave activity [5]. Several works have demonstrated that waking EEG-based spectral variables are influenced by both the circadian and the increased homeostatic modulations, showing that the EEG experiences a circadian fluctuation in addition to the homeostatic wake-time-dependent rise [7,68]. Our results seemed to be consistent with these findings: whereas significant TE decreases fluctuated along the 36 h, significant TE increases appeared mainly between 16 h and 28 h. indicating that the need for sleep was not powerful enough to show statistically significant changes in the first 20 h (see Figure 3). From this point on, the incidence of homeostatic component increased and it was more prominent in relation to the circadian process. 

As discussed above, generalized TE decreases and a local increase of TE in the frontal area were found during prolonged wakefulness. Interestingly, TE decreases showed a very similar course over time to theta power, exhibiting significant changes at the same time points during prolonged wakefulness. Decreases in TE during sleep deprivation reflected both the circadian and the increased homeostatic effects of sleep regulation. Thus, whole-brain TE decreases showed a high correlation (0.932) with theta time course but a poor correlation (0.345) with the subjective somnolence, as measured by a visual analog scale. Conversely, localized frontal TE increases showed a strong correlation (0.721) with the subjective somnolence but a very weak correlation (0.083) with theta activity. Hence, the time course of the frontal TE increases did not seem to be affected by the circadian modulation but the sleep pressure.

Somnolence has been strongly associated with the increase of theta activity in the prefrontal cortex, and frontal theta EEG activity correlates negatively with the DMN in resting state as well as with working memory maintenance [69]. It is also known that amplitude increases in low-frequency oscillations are related to decreased BOLD signals [70]. Subjective sleepiness and spectral electrophysiological variables were highly correlated with TE variables showing that predictive information transfer could be a useful marker for sleep propensity, with the additional property of directed relationships.

Neurophysiology of somnolence is still unclear, although acute and chronic daytime sleepiness might be associated with decreasing functional connectivity in the DMN [71], reflecting a local sleep phase whereby some areas of the brain might be in a sleep state during consciousness [72]. Other recent studies have shown that the pattern of cerebral activity after acute sleep restriction is highly dependent on level of drowsiness. Specifically, drowsy subjects showed reductions in cerebral blood flow of fronto-parietal brain regions, in contrast to non-drowsy subjects who maintained their activity in these attentional regions and also showed an increased activity in the arousal-promoting brain regions as a compensatory mechanism to maintain alertness [73].

Patterns of activation in fronto-parietal areas that overlap across different cognitive functions have been reported, especially those located in the prefrontal areas and sensitive to sleep loss. Accordingly, it has been suggested that the medial prefrontal cortex, one of the cores of the DMN, would be an active part also in the fronto-parietal “mediator” network, an attention mechanism responsible for modulation between internal attentional processes such as memory (DMN) and external stimuli and sensory representations (dorsal attention network) [74].

The increase in anterior TE is in agreement with the double dissociation in the DMN after sleep deprivation commented above, and could support the relevance of this fronto-parietal network for mediate planning between intrinsically and extrinsically driven brain states in a sleep deprivation situation. Moreover, increases in anterior TE indicate an increase in the predictability of cerebral activity in anterior areas, possibly indicating the increasing demands of top-down control attentional and arousal promoting mechanisms. This could reflect the compensatory effort required to offset the homeostatic and circadian drive to sleep when there is no interference from task demands to maintain alertness.

Some limitations of this research merit further consideration. The experimental paradigm affected the interpretation of the results, and in particular, the polysomnographic recordings (PSG) of the preceding and following recovery nights (to the prolonged wakefulness period) would be useful for assessing sleep homeostasis. The study of the correlation between changes in sleep EEG and sleep parameters could help in the assessment of a potential relationship between TE during wakefulness and sleep intensity. Also, no waking EEG recordings were performed after a recovery period, so it was not possible to evaluate whether changes in TE were partially or completely reversed after the dissipation of sleep pressure. Further studies should take into account complementary objective behavioral indexes of cognitive performance, alertness, and sleep propensity for the correlation analysis, and might also include more accurate anatomical mapping such as that provided by simultaneous fMRI to complement the feasibility and reliability of TE as a marker of sleep pressure. Moreover, additional works are also necessary to evaluate the role of TE during the recovery night after a sustained wakefulness, to assess its consistency in the modelling of the reduction of sleep pressure during sleep.

The objective of this study was to assess TE and evaluate its reliability as a possible marker of sleep propensity, and the results demonstrated that decreases during sleep deprivation matched the increases in theta activity and decreases in alpha power and global coherence previously reported [7,8,9,12,24]. Additional topographic information revealed that local TE increases in the frontal region correlated with subjective somnolence, which could be considered a potential measure of sleep pressure.

Insufficient sleep is a very common and increasing problem in our society with important health and quality of life consequences. Although the detrimental effects of sleep loss can differ across subjects, the lack of sleep increases drowsiness and the tendency to fall asleep during the day. This alters not only performance, but can also affect seriously individual and public safety. Controversies exist between subjective and objective measures of drowsiness and alertness, and TE is a measure that could help understand the dynamics in neuromodulatory and connectivity changes that sleep loss provokes in the brain, to advance in the understanding and the minimization of the associated neurobehavioral consequences.

## 5. Conclusions

The results of the present study suggest that TE is an objective connectivity index that could be associated with a potential measure of sleep propensity and somnolence during sustained wakefulness, particularly the connectivity increases found in the anterior area. Moreover, and although further evaluation is necessary, TE could be also useful as a possible predictive index for the assessment of the resilience to psychomotor vigilance impairment during sleep deprivation, or to estimate the vulnerability to psychomotor vigilance decline following sleep deprivation.

## Figures and Tables

**Table 1:** Color and line code for statistical difference representation in maps.

Probability Value (*p*)	Color Intensityof the Electrode	Color and Thickessof the Connection
*p* ≤ 0.01	dark	dark, thick
0.01 < *p* ≤ 0.05	medium	light, thick
0.05 < *p* ≤ 0.10	light	light thin

## Abbreviations

The following abbreviations are used in this manuscript:

ECG	Electrocardiogram
ECG	Electroencephalogram, electroencephalography, electroencephalographic
DMN	Default mode network
MSC	Magnitude squared coherence
*P*	probability value
SD	Sleep deprivation
TE	Transfer entropy
